# Efficacy of AC‐T Versus TAC Neoadjuvant Chemotherapy for Locally Advanced Breast Cancer: A Retrospective Analysis

**DOI:** 10.1155/ijbc/6630563

**Published:** 2026-06-19

**Authors:** Mehrzad Mirzania, Fatemeh Anari, Seyed Amir Hossein Emami, Kamran Roudini, Mehdi Karimi, Kianmehr Saleh, Masoud Mortezazadeh, Ahmad Khajeh-Mehrizi

**Affiliations:** ^1^ Department of Hematology and Medical Oncology, Cancer Institute, Imam Khomeini Hospital Complex, Tehran University of Medical Sciences, Tehran, Iran, tums.ac.ir; ^2^ Faculty of Medicine, Bogomolets National Medical University (NMU), Kyiv, Ukraine; ^3^ Cancer Research Center, Hamadan University of Medical Science (UMSHA), Hamadan, Iran

**Keywords:** anthracycline-taxane regimens, breast cancer, metastasis, neoadjuvant chemotherapy, oncology, pathologic complete response, survival

## Abstract

**Purpose:**

Locally advanced breast cancer (LABC) remains a major therapeutic challenge, with neoadjuvant chemotherapy (NAC) serving as a cornerstone of management. However, direct evidence comparing the efficacy of sequential (AC‐T) and concurrent (TAC) anthracycline–taxane regimens in real‐world Middle Eastern populations remains scarce. This study is aimed at comparing the clinical outcomes of AC‐T and TAC in Iranian women with LABC.

**Methods:**

A retrospective cohort study was conducted among 118 patients with Stage III breast cancer treated at Tehran, Iran. Patients received either AC‐T (doxorubicin/cyclophosphamide followed by paclitaxel; *n* = 70) or TAC (docetaxel/doxorubicin/cyclophosphamide; *n* = 48). Outcomes included pathological complete response (pCR), overall survival (OS), disease‐free survival (DFS), distant recurrence‐free survival (DRFS), and locoregional recurrence‐free survival (LRFS).

**Results:**

Baseline demographic and clinical characteristics were comparable between groups. No significant difference in pCR rates was observed between the groups. The AC‐T regimen showed higher, though nonsignificant, OS at 3, 5, and 10 years (90%, 82%, and 71%, respectively) compared with TAC (83%, 74%, and 57%). Conversely, TAC demonstrated significantly superior 10‐year DRFS (62% vs. 45%, *p* = 0.019). DFS and LRFS were similar between regimens. Multivariable Cox regression analysis identified ER positivity as the only independent predictor of improved OS (HR = 0.42, 95% CI: 0.29–0.82, *p* = 0.012) and DFS (HR = 0.36, 95% CI: 0.18–0.73, *p* = 0.005), whereas the chemotherapy regimen was not independently associated with survival after adjustment for confounders.

**Conclusion:**

Both AC‐T and TAC provided effective neoadjuvant control in LABC, with comparable survival outcomes. These findings support individualized regimen selection based on patient profile and tumor biology. Prospective studies are needed to refine region‐specific treatment strategies.

## 1. Introduction

Breast cancer remains the most frequently diagnosed cancer in women and a leading cause of cancer‐related deaths worldwide. GLOBOCAN 2022 recorded 2.3 million breast cancer diagnoses and 666,103 deaths worldwide—figures that translate to almost one in every four female cancers [1]. Yet these headline numbers conceal striking disparities: although age‐standardized incidence peaks in high‐income countries, over three‐fifths of global breast cancer deaths occur in low‐ and middle‐income countries, reflecting inequities in early detection, treatment access, and continuity of care [2, 3]. Locally Advanced Breast Cancer (LABC), defined as Stage III disease without distant metastases, remains a therapeutically complex entity, characterized by large primary tumors, substantial regional nodal involvement, and a propensity for recurrence that persists even after aggressive multimodality treatment aimed at cure [4].

In the last two decades, neoadjuvant chemotherapy (NAC) has shifted from a salvage measure in inoperable disease to a routine component of care for operable but high‐risk breast cancer [5]. Beyond its cytoreductive role—shrinking primary tumors to permit breast conservation—it can also limit the extent of axillary intervention, reducing lymphoedema risk, and begin systemic micrometastatic control from the outset [6]. Equally important, NAC allows clinicians to observe the tumor′s chemosensitivity in real time, with pathological complete response (pCR) emerging as a surrogate for improved long‐term outcomes in selected subtypes [5]. Among the regimens deployed most widely, AC‐T (doxorubicin and cyclophosphamide, then paclitaxel) is given sequentially, whereas TAC (docetaxel, doxorubicin, and cyclophosphamide) is delivered concurrently—two strategies endorsed by major guidelines yet differing in cycle structure, cumulative doses, and toxicity patterns [7, 8]. Of note, although recent NCCN guidelines have preferentially recommended sequential AC‐T or nonanthracycline TC (docetaxel and cyclophosphamide) regimens, TAC remains an endorsed neoadjuvant option in other international frameworks, including the ESMO Clinical Practice Guidelines and the St. Gallen Consensus, particularly in resource‐limited settings and regions with differing patient demographics where concurrent regimens offer logistical and cost advantages.

Direct regimen‐to‐regimen comparisons have produced mixed results. Some studies describe similar pCR rates between AC‐T and TAC, but divergent side‐effect profiles—more neutropenic fever with TAC and more neuropathy with sequential AC‐T [9, 10]. Survival endpoints such as overall survival (OS), disease‐free survival (DFS), distant recurrence‐free survival (DRFS), and locoregional recurrence‐free survival (LRFS) likewise lack consensus, with reported advantages often shifting according to follow‐up duration and patient characteristics [11]. Importantly, there is a paucity of robust, population‐specific real‐world evidence from Middle Eastern and broader Asian settings, where genetic backgrounds, tumor biology, and resource availability differ from those in Western contexts. Insights from community cancer center studies highlight that care delivery models, patient comorbidities, and access to multidisciplinary oncology services can significantly affect NAC outcomes in everyday clinical practice [12]. Retrospective real‐world analyses in these populations are especially scarce, leaving the decision between simultaneous and sequential anthracycline–taxane therapy for LABC without conclusive regional evidence.

Although both AC‐T and TAC are established NAC regimens for LABC, evidence comparing their efficacy and safety remains inconsistent, particularly in Middle Eastern populations, where treatment outcomes may differ due to genetic and healthcare variations. Limited regional data hinder evidence‐based regimen selection. This study, therefore, is aimed at comparing the efficacy of AC‐T and TAC neoadjuvant regimens in women with LABC treated at a tertiary cancer center in Tehran, Iran, to generate real‐world insights relevant to local clinical practice.

## 2. Methods

### 2.1. Study Design and Setting

This retrospective cohort study was conducted at *Imam Khomeini Hospital*, Tehran, Iran, a tertiary referral center for oncology care. The study period spanned from March 2012 to March 2018, and all data were analyzed up to the latest follow‐up available in the inpatient records. The target population comprised women with histologically confirmed LABC who received NAC with either the AC‐T regimen or the TAC regimen during the study period. Patients were identified through the institutional cancer registry and cross‐verified with the hospital′s electronic medical records. Treatment allocation was not randomized; rather, the choice between AC‐T and TAC was made by the treating medical oncologist based on patient‐specific factors including tumor characteristics, performance status, cardiac function, comorbidities, and patient preference after discussion of the respective toxicity profiles. Both regimens were considered standard of care at our institution during the study period, and no formal institutional algorithm dictated regimen selection.

### 2.2. Eligibility Criteria

The inclusion criteria were as follows: (1) female patients diagnosed with Stage III breast cancer according to the American Joint Committee on Cancer (AJCC) classification at the time of diagnosis; (2) receipt of either the AC‐T or TAC regimen as NAC; and (3) availability of complete baseline demographic, clinical, pathological, and therapeutic data within the institutional archives. These criteria ensured a uniform cohort with comparable baseline and treatment characteristics.

The exclusion criteria were applied to eliminate confounding factors and ensure the internal validity of the study. Patients were excluded if they had: (1) a histological diagnosis other than LABC; (2) received prior adjuvant chemotherapy for breast cancer; (3) been treated with chemotherapy regimens other than AC‐T or TAC; (4) incomplete or missing key treatment or follow‐up data; (5) an age greater than 65 years; (6) an inability to tolerate the complete chemotherapy protocol; (7) received dose‐dense variations of the regimens; or (8) male sex. These exclusions were implemented to maintain a homogenous sample representative of the typical LABC population receiving standard neoadjuvant therapy.

### 2.3. Data Collection

Demographic and baseline clinical variables were extracted, including age at diagnosis, date of cancer diagnosis, tumor stage, and receptor status (estrogen receptor [ER], progesterone receptor [PR], and human epidermal growth factor receptor 2 [HER2]), as well as comorbidities documented before initiation of chemotherapy. Treatment details, including chemotherapy regimen, cycle numbers, dosing adjustments, and delays, were recorded. Follow‐up data captured through outpatient clinic notes, imaging, and pathology reports included recurrence events, presence of distant metastases, and death from breast cancer. These were used to calculate OS, DFS, local recurrence‐free survival (LRFS), and DRFS. pCR was defined as the absence of residual invasive tumor in both breast and axillary lymph nodes on final surgical pathology, regardless of residual ductal carcinoma in situ.

### 2.4. Treatment Protocols

In the AC‐T group, patients received doxorubicin (60 mg/m^2^) and cyclophosphamide (600 mg/m^2^) intravenously every 3 weeks for four cycles, followed by paclitaxel (175 mg/m^2^) every 3 weeks for four additional cycles. In the TAC group, patients received docetaxel (75 mg/m^2^), doxorubicin (50 mg/m^2^), and cyclophosphamide (500 mg/m^2^) intravenously every 3 weeks for six cycles. Growth factor support with granulocyte colony‐stimulating factor (G‐CSF) and antiemetic prophylaxis was administered according to institutional protocols.

### 2.5. Outcome Measures

The primary outcome was the rate of pCR in each treatment group. Secondary outcomes included OS, DFS, LRFS, DRFS, and treatment‐related adverse events. OS was calculated from the date of diagnosis to the date of death from any cause or the last follow‐up. DFS was defined as the time from diagnosis to the first documented local recurrence, distant metastasis, or death. LRFS and DRFS were calculated similarly, restricted to local or distant relapse events, respectively.

### 2.6. Ethical Considerations

The study protocol was approved by the Institutional Review Board of Tehran University of Medical Sciences (Approval No. IR.TUMS.IKHC.REC.1402.096) and performed in accordance with the Declaration of Helsinki. Given the retrospective design and use of anonymized archival data, the ethics committee waived the need for informed consent.

### 2.7. Statistical Analysis

All analyses were conducted using SPSS software Version 16.0 (SPSS Inc., Chicago, Illinois, United States). Categorical variables were summarized as frequencies and percentages, whereas continuous variables were presented as means with standard deviations (±SD) or medians with interquartile ranges, as appropriate. Between‐group comparisons for categorical variables were made using the chi‐square test or Fisher′s exact test. In contrast, continuous variables were compared using the independent‐samples *t*‐test or the Mann–Whitney *U* test, depending on the normality of the distribution. Survival outcomes were evaluated using the Kaplan–Meier method, with differences assessed via the log‐rank test. To identify independent predictors of survival and account for potential confounding variables, a multivariable Cox proportional hazards regression analysis was performed. The model included chemotherapy regimen, Ki‐67 expression, HER2, ER, PR, and tumor grade. A two‐sided *p* value < 0.05 was considered statistically significant.

## 3. Results

### 3.1. Patient Characteristics

The patient cohort consisted of 118 individuals with LABC, distributed between the AC‐T (*n* = 70) and TAC (*n* = 48) treatment groups. Across both groups, there were no statistically significant differences in baseline characteristics, including age, weight, height, body mass index (BMI), systolic and diastolic blood pressure, marital status, or clinical T and N staging, suggesting comparability between the two cohorts. The mean age was slightly higher in the AC‐T group (49.04 ± 11.86 years) compared with the TAC group (45.27 ± 9.14 years), and a similar trend was observed in BMI; however, these differences did not reach statistical significance (*p* > 0.05). The majority of participants were married (over 88% in both groups). Overall, Table 1 demonstrates that patient characteristics were balanced between treatment groups, minimizing potential confounding factors.

**Table 1:** Characteristics of the patients with LABC in the AC‐T (*n* = 70) and TAC (*n* = 48) treatment groups.

Variables	Unit	Total (*n* = 118)	AC‐T groups (*n* = 70)	TAC groups (*n* = 48)	*p*
Age	(Year)	47.50 ± 10.94	49.04 ± 11.86	45.27 ± 9.14	0.069 ^∗^

Weight	(kg)	72.59 ± 12.56	73.40 ± 12.16	71.42 ± 13.17	0.409 ^∗^

Height	(cm)	160.22 ± 5.26	159.94 ± 5.32	160.63 ± 5.21	0.488 ^∗^

BMI	(kg/cm^2^)	28.38 ± 4.84	28.88 ± 5.00	27.65 ± 4.57	0.184 ^∗^

SBP	(mmHg)	117.02 ± 8.08	118.00 ± 8.32	115.65 ± 7.57	0.132 ^∗^

DBP	(mmHg)	74.27 ± 5.34	74.69 ± 5.43	73.69 ± 5.21	0.335 ^∗^

Marital status	Single	11 (9.33%)	8 (11.4%)	3 (6.3%)	0.504 ^∗∗^
	Married	107 (90.6%)	62 (88.5%)	45 (93.8%)	

Clinical T	T1	19 (16.1%)	13 (18.6%)	6 (12.5%)	0.724 ^∗∗^
	T2	46 (41.5%)	30 (42.9%)	19 (39.6%)	
	T3	38 (32.2%)	21 (30%)	17 (35.4%)	
	T4	12 (10.2%)	6 (8.6%)	6 (12.5%)	

Clinical N	N0	27 (22.9%)	20 (28.6%)	7 (14.6%)	0.333 ^∗∗^
	N1	36 (30.5%)	20 (28.6%)	16 (33.3%)	
	N2	45 (38.1%)	24 (34.3%)	21 (43.8%)	
	N3	10 (8.5%)	6 (8.6%)	4 (8.3%)	

*Note:* Data are presented as the mean ± SD for quantitative variables. For categorical variables, the related data are presented as frequency (percent).

Abbreviations: BMI, body mass index; DBP, diastolic blood pressure; SBP, systolic blood pressure.

^∗^Statistical analysis was done by an independent sample *t*‐test.

^∗∗^Statistical analysis was done by the chi‐square test.

### 3.2. Immunohistochemical (IHC) Profiles and Molecular Subtypes

Analysis of IHC‐based receptors revealed no statistically significant differences in Ki‐67 expression, HER2, ER, and PR status between the AC‐T and TAC groups. The mean Ki‐67 expression was slightly higher in the AC‐T group (33.01 ± 31.69) compared with the TAC group (28.12 ± 18.30), although the difference was not significant (*p* = 0.343). Stratification of Ki‐67 by cutoff values (≤10% vs. >10%) revealed a greater proportion of high Ki‐67 expression (>10%) across both treatment groups (88.1% overall), with no significant group variation (*p* = 0.147). HER2 positivity was observed in 31.4% of AC‐T patients and 35.4% of TAC patients (*p* = 0.278), whereas HER2 negativity predominated in both groups. For hormone receptor (HR) expression, a significant difference was identified (*p* = 0.033), with higher positivity in the AC‐T group (74.3%) compared with the TAC group (56.3%). PR expression did not differ significantly (*p* = 0.275), being positive in 61.4% of AC‐T patients and 54.2% of TAC patients (Table 2).

**Table 2:** Comparison of the immunohistochemical profiles and clinicopathological outcomes between the AC‐T (*n* = 70) and TAC (*n* = 48) treatment groups.

Variables		Total (*n* = 118)	AC‐T group (*n* = 70)	TAC group (*n* = 48)	*p*
Ki‐67		31.01 ± 27.04	33.01 ± 31.69	28.12 ± 18.30	0.343 ^∗^

Ki‐67	<10%	14 (11.9%)	6 (8.6%)	8 (16.7%)	0.147 ^∗∗^
	≥10%	104 (88.1%)	64 (91.4%)	40 (83.3%)	

HER2	Negative	81 (68.64%)	50 (71.4%)	31 (64.6%)	0.278 ^∗∗^
	Positive	37 (31.36%)	20 (28.6%)	17 (35.4%)	

ER	Negative	39 (33.05%)	18 (25.7%)	21 (43.8%)	**0.033** ^∗∗^
	Positive	79 (66.95%)	52 (74.3%)	27 (56.3%)	

PR	Negative	49 (41.52%)	27 (38.6%)	22 (45.8%)	0.275 ^∗∗^
	Positive	69 (58.47%)	43 (61.4%)	26 (54.2%)	

Tumor grade	I	3 (2.54%)	2 (2.9%)	1 (2.1%)	0.813 ^∗∗^
	II	84 (71.18%)	50 (71.4%)	34 (70.8%)	
	III	31 (26.27%)	18 (25.7%)	13 (27.1%)	

Pathologic response	Complete	34 (28.8%)	19 (27.1%)	15 (31.3%)	0.632 ^∗∗^
	Noncomplete	84 (71.2%)	51 (72.9%)	33 (68.7%)	

Metastasis	Negative	80 (67.79%)	49 (70.0%)	31 (64.6%)	0.337 ^∗∗^
	Positive	38 (35.31%)	21 (30.0%)	17 (5.4%)	

Recurrence	Negative	105 (88.98%)	62 (88.6%)	43 (9.6%)	0.556 ^∗∗^
	Positive	13 (11.02%)	8 (11.4%)	5 (10.4%)	

Survival	Death	34 (28.8%)	16 (22.9%)	18 (37.5%)	0.065 ^∗∗^
	Alive	84 (71.2%)	54 (77.1%)	30 (62.5%)	

*Note:* Data are presented as the mean ± SD for quantitative variables. For categorical variables, the related data are presented as frequency (percent). The bold value is statistically significant.

Abbreviations: ER, estrogen receptor; HER2, human epidermal growth factor receptor 2; PR, progesterone receptor.

^∗^Statistical analysis was used by an independent sample *t*‐test.

^∗∗^Statistical analysis was used by the chi‐square test.

### 3.3. Clinicopathological Outcomes

In terms of clinicopathological outcomes, the distribution of tumor grades was comparable between groups, with the majority of cases classified as Grade II (71.18% overall, *p* = 0.813). The overall pCR rate was 28.8% in the entire cohort. In the AC‐T group, pCR was achieved in 19 (27.1%) patients compared with 15 (31.3%) in the TAC group, and this difference was not statistically significant (*p* = 0.63). No significant differences were observed in metastasis (*p* = 0.337) or recurrence (*p* = 0.556) rates. Metastasis was present in 35.3% of patients, whereas recurrence occurred in 11.0%. Survival analysis indicated a higher mortality in the TAC group (37.5%) compared with AC‐T (22.9%), although the difference approached but did not reach statistical significance (*p* = 0.065) (Table 2).

### 3.4. OS

**Figure 1:**
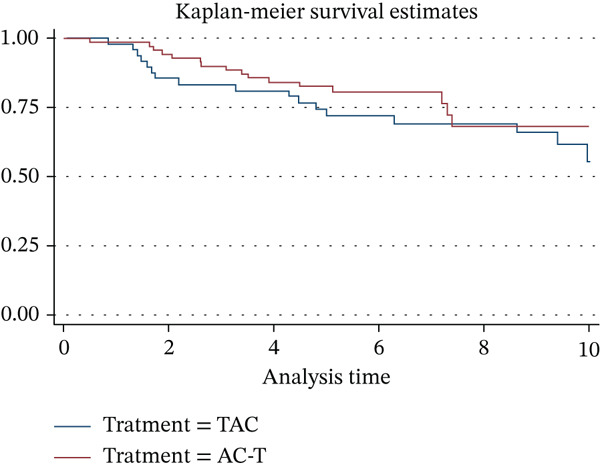
Kaplan–Meier estimates of 10‐year overall survival (OS) by treatment group.

The analysis of OS rates demonstrated that patients treated with the AC‐T regimen had slightly higher survival rates compared with those receiving the TAC regimen, although the differences were not statistically significant (Table 3). The 3‐year OS was 90% in the AC‐T group versus 83% in the TAC group (*p* = 0.069). Similarly, the 5‐year OS was 82% for AC‐T and 74% for TAC (*p* = 0.097). At 10 years, survival declined to 71% in the AC‐T group and 57% in the TAC group (*p* = 0.069). Despite the gradual decline over time, the trend suggested a modestly better long‐term outcome among patients treated with AC‐T, though this did not reach statistical significance. The Kaplan–Meier curve for 10‐year OS across different treatments is presented in Figure 1.

**Table 3:** Overall survival (OS), disease‐free survival (DFS), distant recurrence‐free survival (DRFS), and locoregional recurrence‐free survival (LRFS) of total patients (*n* = 118), AC‐T group (*n* = 70), and TAC group (*n* = 48).

	Total (*n* = 118)	AC‐T group (*n* = 70)	TAC group (*n* = 48)	*p*
Overall survival (OS)				
3‐year OS	87%	90%	83%	0.069
5‐year OS	78%	82%	74%	0.097
10‐year OS	65%	71%	57%	0.069
Disease‐free survival (DFS)				
3‐year DFS	87%	90%	83%	0.77
5‐year DFS	78%	80%	74%	0.65
10‐year DFS	50%	26%	44%	0.25
Distant Recurrence‐Free Survival (DRFS)				
3‐year DRFS	89%	91%	87%	0.72
5‐year DRFS	81%	81%	80%	0.58
10‐year DRFS	51%	45%	62%	0.019
Locoregional recurrence‐free survival (LRFS)				
3‐year LRFS	87%	91%	81%	0.85
5‐year LRFS	78%	81%	72%	0.99
10‐year LRFS	52%	46%	59%	0.49

### 3.5. DFS

**Figure 2:**
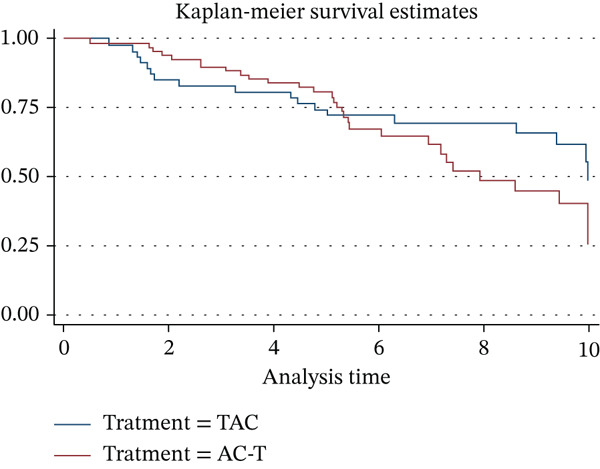
Kaplan–Meier estimates of 10‐year disease‐free survival (DFS) by treatment group.

Regarding DFS, both treatment groups exhibited comparable outcomes (Table 3). The 3‐year DFS was 90% in the AC‐T group and 83% in the TAC group (*p* = 0.77), whereas the 5‐year DFS remained similar at 80% and 74%, respectively (*p* = 0.65). At the 10‐year mark, DFS decreased to 26% for AC‐T and 44% for TAC (*p* = 0.25). Although there was a numerical difference favoring the TAC regimen at 10 years, it was not statistically significant. Kaplan–Meier curves for DFS at 3, 5, and 10 years under different treatment regimens are presented in Figure 2. Overall, both regimens maintained satisfactory disease control during the early follow‐up years, with divergence appearing only in the longer‐term outcomes.

### 3.6. DRFS

**Figure 3:**
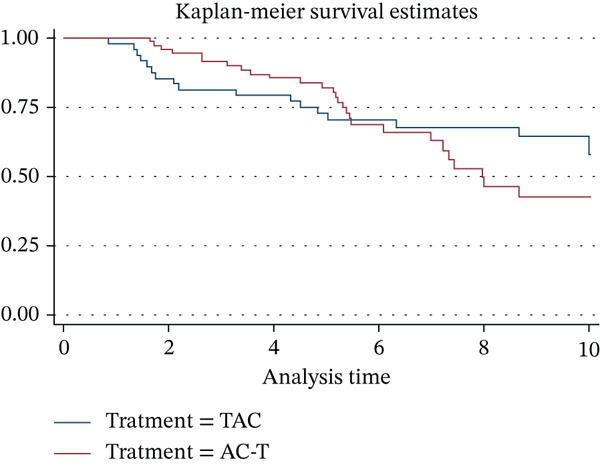
Kaplan–Meier estimates of 10‐year distant recurrence‐free survival (DRFS) by treatment group.

DRFS results indicated no significant difference between groups in the short and intermediate terms but revealed a significant difference at long‐term follow‐up (Table 3). At 3 years, DRFS was 91% for AC‐T and 87% for TAC (*p* = 0.72), and at 5 years, it remained nearly identical at 81% and 80% (*p* = 0.58). However, at 10 years, DRFS decreased to 45% in the AC‐T group compared with 62% in the TAC group, and this difference reached statistical significance (*p* = 0.019). This suggests that patients treated with the TAC regimen experienced better long‐term distant recurrence control than those treated with AC‐T. Kaplan–Meier curves for 10 years of DRFS in both treatment regimens are presented in Figure 3.

### 3.7. LRFS

**Figure 4:**
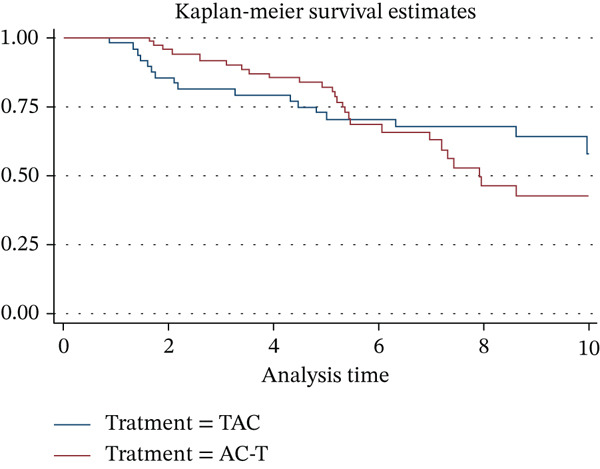
Kaplan–Meier estimates of 10‐year locoregional recurrence‐free survival (LRFS) by treatment group.

The LRFS was comparable between the two treatment regimens across all follow‐up periods (Table 3). The 3‐year LRFS was 91% in the AC‐T group and 81% in the TAC group (*p* = 0.85). The 5‐year LRFS values were 81% for AC‐T and 72% for TAC (*p* = 0.99), and by 10 years, LRFS declined to 46% and 59%, respectively (*p* = 0.49). Despite minor variations, none of the differences reached statistical significance, indicating that both regimens offered similar efficacy in preventing locoregional recurrence over time. Kaplan–Meier curves for 10 years of LRFS in both treatment regimens are presented in Figure 4.

### 3.8. Multivariable Cox Regression Analysis of Predictors for OS and DFS

To identify independent predictors of survival and account for potential confounding variables, a multivariable Cox proportional hazards regression analysis was performed. The model included chemotherapy regimen (AC‐T vs. TAC), Ki‐67 expression (>10% vs. ≤10%), HER2 status, ER status, PR status, and tumor Grade (I, II, III). The results are summarized in Table 4. For OS, ER positivity was the only statistically significant independent predictor of improved survival (HR = 0.42, 95% CI: 0.29–0.82, *p* = 0.012). indicating a 58% reduction in the hazard of death among ER‐positive patients compared with ER‐negative patients. Regarding DFS, ER positivity similarly emerged as a strong independent predictor of favorable outcomes (HR = 0.36, 95% CI: 0.18–0.73, *p* = 0.005), conferring a 64% reduction in the hazard of recurrence or death. These findings indicate that after adjusting for key confounders, the chemotherapy regimen was not independently associated with survival, whereas ER status was a robust predictor of both OS and DFS.

**Table 4:** The association of chemotherapy regimens, the expression of Ki‐67, HER2, ER, PR, and tumor grade on overall survival and disease‐free survival.

Variables		Overall survival			Disease‐free survival		
		HR	CI 95%	*p*	HR	CI 95%	*p*
Chemotherapy regimen^a^		0.56	0.33–1.29	0.222	0.65	0.51–1.06	0.132
Ki‐67^b^		1.00	0.99–1.01	0.289	1.12	0.83–1.23	0.325
HER2^c^		0.99	0.48–2.04	0.990	0.85	0.54–1.84	0.856
ER^c^		0.42	0.29–0.82	**0.012**	0.36	0.18–0.73	**0.005**
PR^c^		0.57	0.29–1.12	0.102	0.69	0.36–1.56	0.458
Tumor grade	**I**	Ref.	Ref.	Ref.	Ref.	Ref.	Ref.
	**II**	0.53	0.07–4.05	0.547	0.36	0.15–6.07	0.698
	**III**	1.14	0.14–8.78	0.898	1.56	0.89–10.21	0.952

*Note:* Data analysis was performed using Cox regression. The bold value is statistically significant.

^a^The TAC group was considered the reference group.

^b^Ki‐67 > 10% was considered the reference group.

^c^Negative subjects were considered the reference group.

## 4. Discussion

### 4.1. Overview of Findings

This retrospective cohort study compared the clinical efficacy of two commonly used NAC regimens, AC‐T (doxorubicin and cyclophosphamide followed by paclitaxel) and TAC (docetaxel, doxorubicin, and cyclophosphamide), in patients with LABC treated at a tertiary oncology center in Tehran, Iran. Although short‐term outcomes such as pCR, DFS, and LRFS were similar between regimens, notable differences emerged in long‐term endpoints. Specifically, TAC demonstrated a statistically significant advantage in DRFS at 10 years, whereas AC‐T showed a trend toward improved OS and early disease control, though not statistically significant.

### 4.2. Comparison With Previous Literature

The comparable pCR rates between AC‐T and TAC observed here align with major randomized trials and meta‐analyses, suggesting similar short‐term tumor response rates for both regimens. Studies such as the NSABP B‐38 trial found no significant difference in DFS or OS between sequential and concurrent anthracycline–taxane combinations, reinforcing that efficacy is largely equivalent when drug exposure is balanced [9]. However, differences in toxicity and long‐term disease control have been inconsistently reported. In our cohort, AC‐T yielded slightly better OS at 10 years, a finding consistent with reports indicating improved tolerability and completion rates for sequential regimens. Conversely, TAC′s superior DRFS parallels observations in some Asian and European trials that concurrent administration may enhance systemic disease control, potentially due to overlapping cytotoxic mechanisms and higher cumulative taxane exposure.

### 4.3. Interpretation of Survival Outcomes

The multivariable Cox regression analysis further elucidated the relationship between treatment regimens and survival outcomes. After adjusting for key potential confounders including HR status, HER2, Ki‐67, and tumor grade, the AC‐T regimen no longer demonstrated a statistically significant OS advantage over TAC, and the TAC regimen′s numerical DFS advantage was similarly nonsignificant. Notably, ER positivity emerged as the sole independent predictor of improved OS (HR = 0.42, *p* = 0.012) and DFS (HR = 0.36, *p* = 0.005) in the multivariable model.

This finding is clinically meaningful. The AC‐T group had a significantly higher proportion of ER‐positive tumors compared with the TAC group (74.3% vs. 56.3%, *p* = 0.033), indicating that the observed OS trend favoring AC‐T in the unadjusted Kaplan–Meier analysis was likely confounded by the more favorable HR profile of that cohort. ER‐positive tumors have a well‐established better long‐term prognosis due to their less aggressive biology and the availability of effective adjuvant endocrine therapy, which can improve survival even after recurrence. Once this imbalance was accounted for in the multivariable model, the regimen‐specific effect on survival was substantially attenuated and no longer approached significance.

These results highlight the importance of cautious interpretation of unadjusted survival comparisons in retrospective studies where treatment allocation is nonrandom. The divergent unadjusted trends—OS favoring AC‐T and DRFS favoring TAC—likely reflect a complex interplay of tumor biology (especially HR status), treatment efficacy, and potential differences in postrecurrence therapies rather than the inherent superiority of one regimen over the other. Future prospective studies with stratified randomization by molecular subtype and standardized adjuvant therapy protocols are essential to definitively compare these regimens.

### 4.4. Regional and Contextual Implications

This study adds valuable real‐world evidence from a Middle Eastern tertiary cancer center, addressing a notable gap in the literature dominated by Western data. Genetic diversity, environmental exposures, and healthcare access differences can substantially influence treatment outcomes. The similar efficacy of AC‐T and TAC in this Iranian cohort suggests that both regimens remain viable first‐line NAC options in regional practice. Importantly, the results highlight the potential for optimizing patient outcomes through individualized regimen selection based on biological subtype and treatment tolerance rather than defaulting to Western‐centric standards.

### 4.5. Limitations

The relatively small sample size and the retrospective single‐center design are important limitations of this study. The nonrandomized allocation introduces selection bias, and unmeasured confounding variables—including postneoadjuvant systemic therapy, surgical approach, and radiotherapy details—may have influenced the observed outcomes. Differences in HER2‐targeted therapy receipt, endocrine therapy adherence, and the type of definitive surgery (breast conserving vs. mastectomy) or radiation fields could not be accounted for in our analysis due to incomplete data in the retrospective records. These factors are known to impact long‐term survival, particularly in HR‐positive and HER2‐positive subgroups, and their absence from our multivariable model represents a potential source of residual confounding. Furthermore, detailed toxicity data (including CTCAE‐graded adverse events, dose reductions, treatment delays, and completion rates) were not systematically collected in our retrospective database and could not be reliably retrieved from the medical records. This limits our ability to compare the tolerability and compliance profiles of the two regimens within this cohort. Given that toxicity and treatment adherence can impact long‐term outcomes and inform clinical decision‐making, this represents an important gap that should be addressed in future prospective studies. Additionally, the exclusion of patients older than 65 years, whereas intended to reduce confounding from age‐related comorbidities and differential treatment tolerance, may introduce selection bias and limit generalizability to the elderly LABC population. Older patients may have differential tumor biology, treatment completion rates, and competing mortality risks that could influence survival outcomes. Finally, improvements in supportive care and systemic therapies since 2018 may limit generalizability to current treatment standards, particularly in HER2‐positive or triple‐negative disease, where targeted therapies now play pivotal roles.

### 4.6. Clinical and Research Implications

Despite these limitations, the findings hold important clinical implications. Both AC‐T and TAC demonstrated substantial long‐term efficacy in LABC, with no clear superiority of one regimen across all endpoints. The modest OS trend favoring AC‐T and DRFS advantage for TAC suggests that regimen choice should consider patient‐specific factors such as comorbidities, subtype, and tolerance. Furthermore, the strong association between pCR and survival underscores the importance of integrating early response assessment into treatment algorithms. Prospective studies in Middle Eastern populations, ideally stratified by molecular subtype and incorporating modern targeted agents, are warranted to confirm these findings and refine regional treatment guidelines.

## 5. Conclusion

In this real‐world retrospective analysis of locally advanced breast cancer, both AC‐T and TAC NAC regimens demonstrated comparable efficacy in achieving pCR, DFS, and LRFS. Although long‐term DRFS favored TAC, AC‐T exhibited slightly higher OS, though neither difference reached statistical significance. Achieving pCR emerged as a robust predictor of favorable survival outcomes across all endpoints. These findings suggest that both regimens remain effective and viable options for LABC management, with regimen selection best guided by individual patient characteristics, tumor biology, and institutional capabilities. Further large‐scale, prospective studies are needed to validate these results and inform region‐specific treatment optimization strategies.

## Author Contributions

Mehrzad Mirzania contributed to study design, interpretation, manuscript drafting, and preparing the final version of the manuscript. Fatemeh Anari contributed to study design, data gathering and manuscript drafting. Seyed Amir Hossein Emami contributed to study design and preparing the final version of the manuscript. Kamran Roudini contributed to study design and manuscript drafting. Mehdi Karimi and Kianmehr Saleh contributed to data gathering, interpretation and manuscript drafting. Masoud Mortezazadeh contributed to methodology and finalizing of the manuscript. Ahmad Khajeh‐Mehrizi contributed to study design, interpretation and finalizing of the manuscript.

## Funding

No funding was received for this manuscript.

## Conflicts of Interest

The authors declare no conflicts of interest.

## Data Availability

The data that support the findings of this study are available on request from the corresponding author. The data are not publicly available due to privacy or ethical restrictions.
